# Cerebro-Spinal Meningitis

**Published:** 1878-11

**Authors:** J. G. Westmoreland


### CEREBRO-SPINAL MENINGITIS.

BY J. G. WESTMORELAND, M.D.

Human suffering and mortality would be greatly lessened could ambitious, investigating men certainly determine the point where sthenia ends and asthenia begins—the exact difference in symptoms of inflammatory and non-inflammatory diseases. Pathological changes indicative of the process producing them are discovered on internal parts by *post-mortem* examinations, but too late to profit in the treatment. On organs subject to inspection during life, changes occur from diseases which are by some attributed to inflammatory process, while others declare no such action exists. Even such structural changes as are usually the result of acute inflammation, whether discovered in life or *post-mortem*, are often declared to be the result of poisoned blood, and the disease asthenic in character.

Now, one of the greatest difficulties in the way of arriving at correct conclusions is found in the fact that general prostration of the vital energies follows violent inflammation of a controlling vital organ, and of other organs when extensively diseased. The circulation declines, nervous power is weakened, the skin becomes cool, and all the organs perform their functions imperfectly. Usually inflammatory condition to any considerable extent, in even unimportant parts, causes increased activity of the circulation, heat of the surface, flushed face, thirst, etc. Let the brain, the heart, or a considerable portion of both lungs, however, be the subject of inflammatory action, and the general symptoms will be vastly different. Instead of the symptoms just named, I should expect generally to find those of an opposite character, and the more violent and extensive the inflammation of these vital organs, the greater will be the general prostration. In place of mental excitement as found in disease of inferior organs, stupor will more likely be present; and scarcely perceptible circulation at the wrist will take the place of frequent bounding pulsations, such as is found in pleuritis, hepatitis, etc.

These facts and conclusions certainly apply to meningitis, and possibly also to diphtheria. The fact that meningitis prevails as an epidemic, does not disprove its inflammatory charater any more than the occasional prevalence of epidemic pneumonia proves it non-inflammatory. The tendency to inflammation in certain parts, from peculiar atmospheric conditions, or other causes transmitted through the air, must be admitted. Extensive inflammation of the lungs occurs with many in the same neighborhood, often under such circumstances, leading to great prostration, sometimes from the shock to the nervous centers, sometimes from want of proper oxygenation of the blood in the lungs. These facts do not prove the disease non-inflammatory, however completely prostrate the subject may be early in the progress of the disease.

As evidence that depression of the nervous energies does not necessarily depend on blood poison, so-called, we can call to mind the utter prostration that results from extensive traumatic injury, giving immediate and powerful shock to the brain.

If these facts be recognized as true, how much more certainly should we look for prostration when a controlling vital organ, such as the brain and spinal cord, are the subjects of extensive inflammation? Though the envelopes of the nervous centers are the parts primarily implicated, the organs themselves are not only embarrassed by the changes effected in the meninges, but extension of the disease to the centers themselves takes place more or less, no doubt, in all cases.

If we should meet with a case of stupor amounting almost to coma, with cool skin, feeble pulse more or less opisthotonos, and dilated pupil, poisoning of the brain by some narcotic should not be too hastily acknowledged as the cause. Such symptoms are sometimes found early in meningeal inflammation, and are dependent upon the great determination of blood to the cranial cavity, pressing unduly upon the brain, and thus partially destroying its function. Any means which will lessen this excessive amount of blood in the cranium, tends to restore the cerebral function and to restrain the progress of inflammation. Tonics of quinine and other supporting means cannot, of course, change this dangerous condition, and in depending upon them we but pursue an expectant course, which is to result fatally to the patient if the disease is of sufficient violence to produce death.

During the epidemic of 1872, most physicians in Atlanta pursued this course, and concluded that the poison had made such a powerful impression upon the brain that its energy could not be restored. Under these circumstances nothing could be lost by pursuing a different course. Nothing more than death could be the result of any treatment, and a bold innovation could not be looked upon as hazardous beyond the unsuccessful course pursued.

The report of autopsies invariably had shown evidence of acute inflammation of the meninges, and the symptoms being such as might result from such condition, treatment to change this pathological state seemed to be a rational course of procedure. Of course the usual indications for active depletion, such as full, strong pulse, had to be discarded; for sometimes when the physician was first consulted, the pulse was barely perceptible, owing to the pressure upon the brain by accumulation of blood in the cranium. In this state, abstraction of blood can be had with difficulty. From the arm it will flow very slowly, and of course cups do not deplete rapidly. Persistence in both, however, will slowly drain from the head so as to relieve the cerebral oppression and lessen the danger of fatal results of the inflammation.

Syncope is the first evidence of excessive depletion; and it is not likely to occur in meningitis from abstraction of blood. When so completely surcharged with blood it is no easy matter to deprive the brain of a quantity sufficient to produce syncope. Therefore, when cool skin and feeble circulation are the result of cerebral oppression from determination of blood to the organ, no fears need be entertained of increasing the depression by general depletion to a reasonable extent.

